# Fatty Acids of Semi-Hard Cheese Made from Milk of Goats Fed Diets Enriched with Extruded Linseed or Pumpkin Seed Cake

**DOI:** 10.3390/foods11010006

**Published:** 2021-12-21

**Authors:** Željka Klir Šalavardić, Josip Novoselec, Mario Ronta, Dušica Čolović, Marcela Šperanda, Zvonko Antunović

**Affiliations:** 1 Department for Animal Production and Biotechnology, Faculty of Agrobiotechnical Sciences Osijek, Josip Juraj Strossmayer University of Osijek, V. Preloga 1, 31000 Osijek, Croatia; jnovoselec@fazos.hr (J.N.); mronta@fazos.hr (M.R.); marcela.speranda@fazos.hr (M.Š.); zantunovic@fazos.hr (Z.A.); 2 Institute of Food Technology, University of Novi Sad, Car Lazar Blvd. 1, 21000 Novi Sad, Serbia; ivanovdus@yahoo.com

**Keywords:** goat cheese, chemical composition, fatty acids, extruded linseed, pumpkin seed cake

## Abstract

The addition of oilseeds and their cakes to the diets of lactating dairy goats is an alternative to supplemental feeding, which improves the lipid profile of goat cheeses. The objective of the present study was to evaluate the effect of a diet containing extruded linseed or pumpkin seed cake on the fatty acid profile of semi-hard cheese made from goat milk. The research was carried out with 28 French Alpine goats fed the following diets: 1—basal diet based on extruded soybean and soybean meal; 2—basal diet with 90 g/kg DM extruded linseed (ELS); and 3—basal diet with 160 g/kg DM pumpkin seed cake (PSC). Bulk milk from three separated milk tanks at three samplings was used for the manufacture of four traditional semi-hard cheeses from each milk tank at each sampling on the family farm. The ELS and PSC diets increased fat content in the cheese. The ELS feeding increased the proportion of C18:1 c9, C18:2 c9t11, and C18:3 n-3 in cheese and lowered C8:0, C6:0, and C16:0, while PSC resulted in the highest C18:2 n-6 proportions in the cheese. The health-promoting index was the highest in the cheese of ELS. The ELS had a contribution to higher nutritional and health quality of semi-hard traditional goat cheeses, thus representing a food with health-promoting properties.

## 1. Introduction

Goat milk is mainly processed into cheese, and the world’s production of goat cheese is 564,075 t according to the FAO [1] data. Production of high-quality goat cheeses is associated with Mediterranean countries such as France, Italy, and Spain, while in most countries, goat milk is consumed more locally [2]. Numerous nutrients, particularly fat and proteins transferred from milk into cheese, are very important in the manufacturing of traditional goat cheeses [3]. Fatty acids (FAs) of goat milk affect its nutritional quality and consequently the quality of dairy products, directly affecting cheese taste as well [4]. The goat milk aroma contributes to unique organoleptic properties which are critical to the overall quality of goat cheeses [5].

Fat supplementation with beneficial oils in the diet of dairy goats usually promotes a decrease in the proportion of saturated fatty acids (SFAs), such as lauric (C12:0), myristic (C14:0), and palmitic (C16:0), and an increase in the proportion of polyunsaturated (PUFAs) and long-chain fatty acids (LCFAs). It is already known, from many studies, that extruded linseed added in concentrate mixtures for dairy goats is of high nutritive value, especially regarding n-3 FAs in milk or cheese, such as α-linolenic acid (C18:3 n-3, ALA). Soybean meal has been more frequently replaced by alternative sources of plant fat and protein in livestock diets [6], due to its doubtful origin and negative environmental effect [7]. Due to the high content of crude protein (623 g/kg DM) and high concentration of unsaturated FAs, of which oleic (C18:1 c9, OA; 29.6 g/100 g FAs) and linoleic (C18:2 n-6, LA; 49.0 g/100 g FAs) are predominant [7], pumpkin seed cake is good quality feedstuff for small ruminants’ nutrition. According to Zdunczyk et al. [8], pumpkin seed cake contains an even higher concentration of crude proteins than soybean meal, a feedstuff commonly used as a protein source in ruminants’ diets. As reported by Haenlein [9], in addition to taste, goat milk and its products are favoured for their benefits regarding human health and nutrition, due to greater digestibility, healthier lipid profile, and lower allergenic properties compared to cows’ milk. Moreover, goat milk has been proposed to be considered as a nutraceutical [10]. According to Paskaš et al. [11], dairy products made from goat milk should be more clarified as good quality products, as dairy products with added value, or as products with health-promoting benefits, which could lead to better placement on the market and achievement of a higher price. The chemical composition and health aspect of milk and cheese is a result of many effects in which natural health-promoting compounds in feedstuffs play a strategic role that may modulate FA profile without additional fat supplementation, which is worth further study [12].

Given the health and nutritional benefits of goat milk and its products, as well as the importance of the feeding effect, the objective of the present study was to evaluate the effect of feeding dairy goats with diets enriched with extruded linseed or pumpkin seed cake and to research its effect on the fatty acid proportions of semi-hard goat cheese manufactured traditionally on a family farm.

## 2. Materials and Methods

The study was conducted within the legal regulations according to the Animal Protection Act of Croatia (NN 133/06, NN 37/13 and NN 125/13) and within the European Union Directive 2010/63/UE regarding animal protection as approved by the Bioethics Committee for Research on Animals of the Faculty of Agrobiotechnical Sciences, Osijek.

### 2.1. Experimental Design, Animals, and Feeding

The present research was carried out with 28 dairy goats of French Alpine breed at a commercial family farm in Osijek, Croatia (Osijek-Baranya County). Goats had kidded in a period of one week. To allow the rumen to fully adapt to experimental diets, animals were subjected to experiment (12th ± 3 days of lactation) after seven days of adaptation period. This research is part of a larger study from which some part of the milk results was published in Klir et al. [13] and Klir Šalavardić et al. [14]. Duration of the current study was 75 days, with three treatments and three processing periods. The sampling was conducted when goats were in the 32nd, 60th, and 87th (±3 days) days of lactation. Goats were kept together in the barn and machine milked in the morning and evening. All goats were healthy with adequate body condition score (2.9) and average age of 3.6 years when the experiment started. Goats’ feeding was based on red clover and grass hay (ad libitum), and in addition, each goat received 1 kg/day of a concentrate mixture. Goats were fed individually in separated feeding troughs in the milking parlour. All goats had free access to fresh water, and no refusals of concentrate mixtures were observed.

There were three diets used in the experiment (Table 1): 1—basal diet based on extruded soybean and soybean meal; 2—basal diet with 90 g/kg DM of extruded linseed (ELS) partially replacing soybean; and 3—basal diet with 160 g/kg DM pumpkin seed cake (PSC) completely replacing soybean.

### 2.2. Analysis of Feedstuffs

Composition of concentrate mixtures and hay was determined by using standard methodology [15]. The Kjeldahl method was used to determine crude protein concentrations, while the crude fat concentrations were analysed using Universal Extractions System B-811 (Buchi, Switzerland). The fatty acid proportions in feed were determined by methodology of State Office of Agricultural Chemistry in Baden Württemberg (LaChemie P23-5-008, V. 01). Nitrogen-free extract was calculated by using the following equation:

N-free extract (g/kg DM) = 1000 − (crude protein + crude lipid + crude fibre + ash) (1)

### 2.3. Chemical Analysis of Cheese

Forestripping was done just before milking, and none of the goats presented mastitis. Bulk milk from three separated bulk tanks (one per treatment during morning milking) was used for cheese manufacturing. Home-made cheeses were produced in a traditional way on a family farm. Four cheeses, from each set, were manufactured in a mini cheese factory on the farm and maintained 24 h before packaging. Cheesemaking was performed on 32nd, 60th, and 87th day of lactation (20th, 48th, and 75th day of the study period, respectively); therefore, total sample size was 36 chesses comprised of 12 cheeses per group collected during three samplings. Firstly, the milk was cooked until the temperature reached 89 °C, when vinegar was added (100 mL/10 L of milk). The curd was drained, and when the desired moisture was reached, it was distributed into 350 g perforated moulds and left to rest for 24 h at room temperature. Cheese was vacuumed and stored in freezer (−20 °C) until the analyses were carried out. According to International Food Standards [16], the cheeses’ designation was calculated according to moisture on a fat-free basis and classified as semi-hard.

Composition of semi-hard cheese was determined using standard methods [15]. The crude protein content of cheese was estimated by Kjeldahl method (Kjeldahl steam distillation; Behr, Germany). The crude fat content was analysed using the Universal Extractions System B-811 (Buchi, Switzerland). Crude ash concentrations were determined by incinerating the cheese samples for 4 h at temperature of 550 °C. Value of pH was determined with a contact pH meter (Mettler Toledo, Greifensee, Switzerland). The chemical analyses of semi-hard goat cheeses were performed on each cheese in duplicate values.

### 2.4. Fatty Acid Analysis of Cheese

The extraction of the fat phase for FA analysis from the cheese samples was done as described by Folch et al. [17]. Extracted lipids were used to prepare fatty acid methyl esters (FAME) by transmetilation using 14% wt. of boron trifluoride/methanol solution [18,19]. The acid catalysed methylation was carried out in conditions of temperature <50 °C/20 min to prevent CLA isomerization, as recommended by Park et al. [20]. The samples were analysed by Agilent 7890A (Agilent Technologies, Santa Clara, CA, USA) gas chromatographer with flame ionization detector and auto injection module for liquid, equipped with fused silica capillary column (Supelco SP-2560 Capillary GC Column 100 m × 0.25 mm, 0.20 μm film), and helium (He) as a carrier gas (purity: 99.9997 vol. %, flow rate: 1.5 mL/min and pressure: 1.092 bar). The samples were injected (1μL) in split regime (30:1). Initial temperature was 140 °C, while initial temperature hold-time was 5 min, and heating rate was 3 °C/min. The final temperature was 240 °C, and final temperature hold-time was 10 min, while makeup gas was nitrogen (N). The FA peaks were identified when retention times were compared to retention times of the standards used (Supelco 37 component FAME mix) and with data from internal data library, based on previous research and FAME determination on gas chromatography (mass spectrometry). The results were presented as mass of individual FAs or groups of FAs (g) in 100 g of FAs, or as relative mass contents.

According to the determined FAs, atherogenic (AI) and thrombogenic (TI) indices of cheese made of goat milk were calculated following the equations of Ulbricht and Southgate [21], while health-promoting index (HPI) of cheese was calculated as described in Chen et al. [22]:

AI = [C12:0 + (4 × C14:0) + C16:0]/[ Σn-6 +Σn-3 + ΣMUFAs](2)

TI = [C14:0 + C16:0 + C18:0]/[(0.5 × ΣMUFAs) + (0.5 × n-6) + (3 × n-3) + (n-3/n-6)](3)

HPI = (n-3PUFAs + n-6PUFAs + MUFAs)/[C12:0 + (4 × C14:0) + C16:0](4)

where MUFAs means monounsaturated fatty acids.

### 2.5. Statistical Analysis

Average values for cheese composition and FA proportion were obtained for each group of goats and were subjected to an analysis of repeated measure using PROC MIXED procedure and the following the model Yijk = μ + d_i_ + h_ij_ + p_k_ + dp_ik_ + e_ijk_, where μ = overall mean, d_i_ = fixed effect of goats’ diet (i = Control, ELS, PSC), h_ij_ = goats within diet as subject (j = 9–10), p_k_ = effect of lactation period (k = 1–3), dp_ik_ = interaction between diet and period, and eijk = random error variation (residual error). Mean values were compared by using Tukey’s honestly significant difference test, while *p* ≤ 0.05 indicated significance. Pearson’s correlation coefficients were used to determine the association between certain variables. The value of the correlation coefficient (r) and its description were as follows: 0.00–0.19, very weak; 0.20–0.39, weak; 0.40–0.59, moderate; 0.60–0.79, strong; and 0.80–1.0, very strong, as described in Soeharsono et al. [23]. All results were analysed using SAS 9.4 (SAS Institute Inc., Cary, NC, USA).

## 3. Results

**Figure 1:**
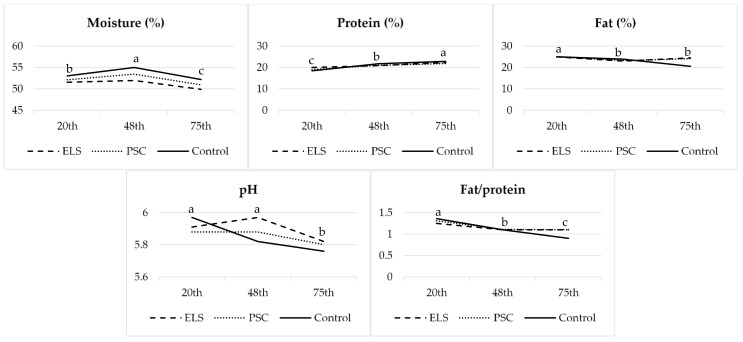
Gross chemical composition of semi-hard cheeses influenced by study period (32nd, 60th, and 87th day of lactation) of goats fed feed mixtures containing extruded linseed or pumpkin seed cake (ELS and PSC, respectively). ^a, b, c^ Values with different superscripts differ significantly at *p* ≤ 0.05.

Table 2 presents the chemical composition of cheese from the milk of goats fed control, ELS, and PSC diets. Goats fed ELS showed the lowest (*p* ≤ 0.05) moisture and a higher pH value than those of other groups. The ELS and PSC increased the fat content of goat cheese compared to the control group (*p* ≤ 0.05). Feeding goats with ELS or PSC did not influence protein or ash content (*p* ≥ 0.128). However, PSC diets increased (*p* ≤ 0.05) the fat/protein ratio in cheese compared to the control group, while ELS did not reflect any changes. Figure 1 presents the gross chemical composition of cheeses influenced by the study period of goats fed feed mixtures containing ELS and PSC. It is evident that moisture content, pH values, and fat/protein ratio decreased (*p* ≤ 0.05), while protein content increased (*p* ≤ 0.05) during lactation. The fat content decreased (*p* ≤ 0.05) from the 20th to 48th days and was very stable at the 48th and 75th days of the study period.

Feeding goats with ELS or PSC influenced the proportions of certain individual FAs and groups of FAs in cheese (Table 3 and Table 4). The proportion of C4:0 in cheese was higher in the ELS compared to the PSC and control (*p* ≤ 0.05), while caprylic (C8:0) and C15:0 were lower in ELS but did not differ in PSC compared to the control. The proportions of caproic (C6:0), C16:0, and C16:1 c9 in cheese were lower in ELS compared to the control or PSC (*p* ≤ 0.05). Both ELS and PSC lowered capric acid (C10:0), C12:0, and C17:0 proportions in cheese compared to the control group. The proportions of C14:0 were the highest in the cheese of the control, followed by PSC and then the ELS group. The proportion of stearic (C18:0) was the highest (*p* ≤ 0.05) in ELS, followed by PSC and then the control. The highest proportions of C18:1 t9, OA, and conjugated linoleic acid (C18:2 c9t11, CLA) were in the ELS group compared to the PSC or control (*p* ≤ 0.05). ELS increased CLA by 25.5% compared to the control group. A higher (*p* ≤ 0.05) LA proportion was found in PSC compared to ELS, while the highest proportions of ALA were found in ELS compared to the control, and the lowest (*p* ≤ 0.05) were in PSC. The ELS increased ALA proportions by 55.7% compared to the control group and by 81.9% compared to PSC. Both ELS and PSC diets lowered C20:4 proportions in cheese. The ELS feeding increased C20:0 and C21:0 compared to the PSC or control group.

Table 4 presents FA groups and calculated indices of cheese from milk of goats fed control, ELS, and PSC diets. The lowest proportions of medium-chain fatty acids (MCFAs) were determined in ELS compared to the PSC and control, while LCFA was higher in ELS compared to PSC, and the lowest was in the control group. The lowest total SFAs were observed in ELS compared to the PSC or control, while proportions of MUFAs were the highest in ELS compared to other groups. Total n-3 proportions in cheese were the highest in ELS, then in the control, and followed by PSC, where the lowest proportions were determined. The n-6/n-3 ratio was the lowest in ELS cheese (decreased by 33.5%), the medium value was in the control, and the highest was in PSC (*p* ≤ 0.05) (increased by 20.7%), compared to the control group. AI was the lowest in cheese of ELS, followed by PSC and then control group, while TI was the lowest in ELS compared to PSC or the control. The health-promoting index was the highest (*p* ≤ 0.05) in cheese of ELS compared to PSC or the control.

**Figure 2:**
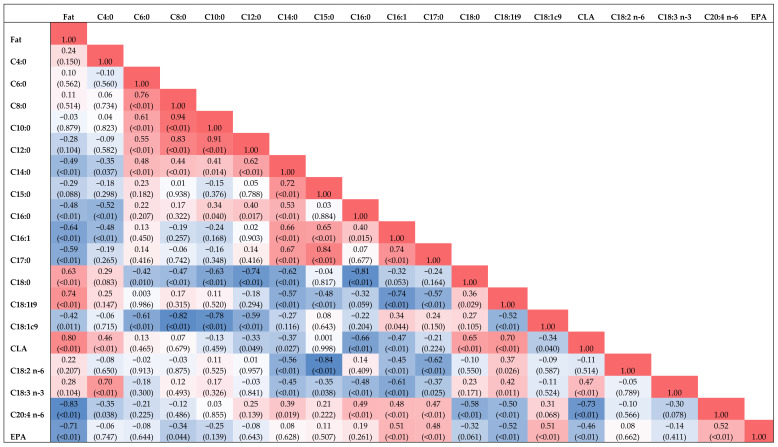
Heatmap of correlations among cheese fat and certain fatty acids, where red means positive correlation, and blue means negative correlation.

There are many correlations among FA proportions in the cheese, as presented in Figure 2. Negative correlations (*p* ≤ 0.05) were found between fat content and C14:0, C16:0, C16:1, C17:0, OA, C20:4, and EPA. Positive correlations (*p* ≤ 0.05) were found between fat and C18:0, C18:1 t9, and CLA. The C18:0 was negatively correlated (*p* ≤ 0.05) with the most individual short-chain fatty acids (SCFAs), as well as MCFAs and LCFAs such as C20:4 and EPA, while a positive correlation was observed with C18:1 t9 and CLA. The OA was negatively correlated (*p* ≤ 0.05) with individual SCFAs, except C4:0, as well as some MCFAs, except C16:1 and C15:0. The LA and ALA were negatively correlated (*p* ≤ 0.05) with most of the MCFAs but were positively correlated with C18:1 t9.

**Figure 3:**
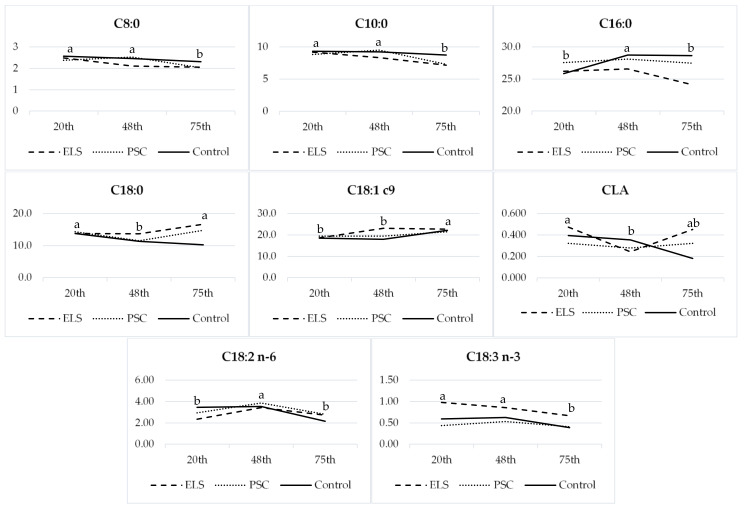
Fatty acids (g/100 g FAME) of semi-hard cheeses influenced by study period (32nd, 60th, and 87th day of lactation) of goats fed feed mixtures containing extruded linseed (ELS) or pumpkin seed cake (PSC). ^a, b, c^ Values with different superscripts differ significantly at *p* ≤ 0.05.

The period influences most of the researched parameters of the chemical composition, such as moisture, proteins, fat, and pH, while interactions between diet and period were determined in proteins, fat, and pH. Most of the FAs were under the influence of the period and interactions between diet and period. Certain FAs in cheese showed a fluctuation during lactation (Figure 3), such as C8:0, C10:0, C16:0, C18:0, OA, CLA, LA, and ALA.

## 4. Discussion

The FA proportion of cheese is strongly affected by the FA proportion in milk [24], which is likewise affected by the goats feeding the most. However, there is a scarcity of detailed information on FA proportion of goat cheeses influenced by feeding strategies. Furthermore, there are several studies on milk FAs, but not its final processed products. As reported by Giorgio et al. [12], different technologies of cheese manufacturing and ripening make comparisons from the literature very difficult. Moreover, the gross composition of cheese is mostly influenced by the technological cheese-making process, while FA profile is mostly related to milk composition [25].

In the present study, an increase in cheese fat is observed in ELS group, which can be attributed to the higher intake of ether extract and fibre content by goats (Table 1 and Table 2), which may have favoured the acetic fermentation in the goats’ rumen. However, similar results regarding fat content in cheese were observed in PSC, although composition of this concentrate mixture was more similar to the control group. When the fat content of cheese decreases, physical and flavour changes lead to products of lower quality [26]. Accordingly, the moisture content was the lowest in the ELS group, followed by PSC and finally the control group, which is in interrelation with the increased fat content in ELS and PSC. The pH value of cheese was higher in ELS compared to the control and PSC. This was similarly determined by Medeiros et al. [27] with the addition of different vegetable oils in the diets of goats, which increased pH values (sesame oil, 5.95; faveleira oil, 6.11; or castor oil, 6.10) compared to the control (5.80), leading to a higher softness and cohesiveness of cheese. However, PSC diets increased (*p* ≤ 0.05) the fat/protein ratio in cheese compared to the control group, while ELS did not reflect any changes. Park et al. [28] observed that total solids in goat milk, especially fat and proteins for hard and semi-hard cheeses, increased as lactation progressed. A similar finding was observed in semi-hard goat cheese of the present study, such as increased protein content and decreased fat/protein ratio, while fat content was very stable during the 48th and 75th days of the study period.

The addition of ELS and PSC had an effect on the proportion of individual SCFAs (C4:0 to C10:0). This could change the sensory properties of cheese because these FAs are responsible for the specific aroma of milk and its derivates [9]. “Goaty” flavour mainly derives from the presence of SCFAs, such as C6:0, C8:0, and C10:0 acid [29], which are not detected in products from cows’ milk [30]. The results of the present study indicated that lowering the proportion of these FAs in the ELS group could possibly be due to a higher proportion of C18:0 in the diet, which is an inhibitor of de novo FA synthesis in the mammary gland, mainly C8:0-C16:0 [31]. However, Gómez-Cortés et al. [32] reported that the acceptability of cheese enriched with beneficial dietary FAs by consumers was similar to cheeses from conventionally fed ewes.

Pasture feeding and fat supplementation in diets have been proven to be a good dietary strategy in small ruminants’ feeding, in order to increase beneficial FAs, among them CLA, vaccenic acid (C18:1 t11), and ALA, which have been shown to possess nutritional properties that reduce cardiovascular risk in humans [33,34]. The inclusion of ELS and PSC in diet caused an increase in the proportion of C18:0 in cheeses, which increased from 11.8 in the control to an average 14.7 in ELS and 13.5 g/100 g FAME in PSC. The increase in C18:0 proportion in cheese can be attributed to a higher C18:0 proportion in diets of goats fed with ELS as reported in Table 1. This seems to be beneficial, as part of C18:0 will be converted into OA by the Δ-9 desaturase enzyme in the mammary gland, which is responsible for the unsaturation of 18:0 at carbon 9, producing OA presented in the final product [35], which was higher in the ELS group compared to the control or PSC in the present study. Innosa et al. [36] did not determine any significant changes in fresh cheese of goats fed diets supplemented with olive leaves, rich in OA. This suggests that OA proportions in cheese are mainly derived from biohydrogenation processes in the rumen and synthesis in the mammary gland on the basis of C18:0. Feeding goats with ELS increased CLA proportions, likely due to the ruminal biohydrogenation process within which the CLA is one of the products. Therefore, feeding ELS may ensure higher CLA proportions in goat cheese, which is already known to have a beneficial effect on human health. Zlatanos et al. [37] reported that the consumption of sheep and goat cheeses within the Mediterranean diet in Greece is much higher than in the rest of the European Union countries and is possibly related to the lower percentage of breast cancer deaths in Greece.

The LA proportion has been reduced in ELS compared to PSC. Gomez-Cortes et al. [32] did not observe any differences in LA proportions in cheese when goats were fed either a low or high level of extruded linseed in the goats’ diets compared to the control. Similarly, no differences were observed in the present study in ELS compared to the control, although it was reduced compared to PSC. The pumpkin seed cake is rich in LA, which is predominant in mixtures containing PSC, as reported in Table 1. It is very well known that linseeds contain a high proportion of ALA, which influences an increase of its proportion in milk and cheese [12,21] if animals are fed with diets containing it in a certain amount. The extrusion process of linseeds may contribute to higher availability of ALA in the digestive tract, which would result in an increased ALA proportion in milk [32] and therefore also in cheese. Similarly, Consetino et al. [38] reported an improved fatty acid profile of Padraccio cheese, from a nutritional perspective, when extruded linseed was implemented in goats’ diets. Consequently, the cheeses from goats fed ELS in our study could be classified as a high source of n-3 FAs under current EU regulations [39]. Consequently, these proportions of LA and ALA resulted in the lowest n-6/n-3 ratio in ELS cheese, the medium value was in the control, and the highest was in PSC. A similarly decreased n-6/n-3 (3.00) ratio was found by Morsy et al. [40] in cheese from goats fed linseed supplementation (50 g/d/head) in their diets. The n-6/n-3 ratio decreased in the present study by over 33% which means that feeding 90 g/d/head of ELS increased a beneficial property of cheese remarkably. This condition is in relation with decreased risks for cancer growth and the reduction of coronary disease risk [41]. This was also proven by decreased AI and TI and increased HPI. Paszczyk and Łuczynska [42] concluded that goat cheeses were characterized by the lowest TI index value, compared to cow and sheep cheeses. As reported by Giorgio et al. [12], dairy products with calculated high values of HPI possibly have more benefits for human health as influenced by certain FAs in cheese, such as PUFAs and CLA having antiatherogenic effects. The opposite trend was observed in this regard for cheese from the PSC group, due to an increase of LA proportions (Table 3). Furthermore, according to abovementioned, ELS feeding contributed to increased proportions of LCFAs, MUFAs, and n-3 and a decrease in SFAs as well.

The highest C18:0 values were observed in cheeses with the highest fat content, which is determined in cheese of ELS goats, as proven by a strong positive correlation between C18:0 and fat content in cheese. Giorgio et al. [12] determined a similar connection between SFAs and fat content in goat cheese. Similarly, CLA proportion has a very strong positive relation with the fat content of cheese in the present study, which is very important for the cheese manufacturing processes, as observed by Zlatanos et al. [37]. These authors observed changes in the CLA proportions which are noticed in cheese during processing, which contains an average of 0.9% of CLA in aged hard cheese made of sheep or goat milk. The results of the present study suggest that increased fat content may be an indicator of increased CLA proportion in cheese under the current experimental setup. The SCFAs, such as C6:0, C8:0, and C10:0, have a moderate to strong negative relation with C18:0 and a strong to very strong negative relation with OA. These LCFAs could suppress the synthesis of de novo FAs in the mammary gland, such as SCFAs, which can be reflected in milk products, such as cheese. The LA and ALA mainly have a moderate negative relation with some MCFAs, such as C14:0 and C16:1, while ALA also has a moderate negative relation with C16:0. Moreover, decreased proportions of C12:0, C14:0, and C16, as well as increased proportions of n-3 (ALA) and MUFAs (OA), in the present study contributed to the higher HPI of cheese intended for human consumption.

Most of the FAs were under the influence of the period and interactions between diet and period. Our study was long-lasting; thus, cheeses were made during different lactation stages, influencing different chemical compositions and FAs. Lactation is a very demanding period for the goats, during which significant changes in metabolism occur [43]. It is already known that milk FAs are connected to changes in energy balance during goats’ lactation [31], which consequently affects cheese FA proportions. These fluctuations during lactation affected the decreased proportions of C8:0 and C10:0 during lactation, while C18:0 and OA increased, which is also consistent with Figure 2, and their interrelations. These fluctuations could reflect an increased uptake of dietary FAs by adipose tissue after the lactation peak of goats [44]. Simultaneously, LA and ALA were decreasing in all groups after the 48th day, while CLA decreased from the 20th to 48th day of the study period.

## 5. Conclusions

Feeding goats with ELS or PSC may affect the gross chemical composition of cheese, especially an increase of fat content. The ELS feeding of goats increased the proportion of beneficial FAs, such as OA, CLA, and ALA, in cheese and simultaneously decreased the C6:0, C8:0, and C10:0 responsible for a particular aroma, while PSC resulted in the highest LA proportion. The n-6/n-3 ratio was the lowest in the ELS cheese with the highest health-promoting index. Moreover, goats fed with mixtures containing ELS derived a health-promoting property for cheese, which also may have commercial relevancy. Our study demonstrated the possibility to apply this experimental feeding with the aim to produce healthier traditional semi-hard goat cheeses on a family farm.

## Figures and Tables

**Table 1:** Ingredient and nutrient composition of control, extruded linseed (ELS), and pumpkin seed cake (PSC) experimental diets.

Ingredient (g/kg DM)	Dietary Treatment		
	Control	ELS	PSC
Corn	429	408	459
Barley	80	80	90
Oat	100	100	135
Wheat flour	120	90	120
Extruded soybean meal	150	-	-
Extruded linseed	-	90	-
Pumpkin seed cake	-	-	160
Alfalfa dehydrated	-	40	-
Soybean meal (46% crude protein)	85	157	-
Calcium carbonate	16	15	16
Monocalcium phosphate	5	5	5
Salt	4	4	4
Pellet binder	1	1	1
Mineral vitamin premix	10	10	10
Nutrients
DM (g/kg fresh matter)	876	874	873
Crude protein (g/kg DM)	162	162	163
Crude fibre (g/kg DM)	41.4	48.8	37.3
Crude ash (g/kg DM)	49.2	50.6	52.3
Ether extract (g/kg DM)	56.4	58.3	56.3
Nitrogen free extract (g/kg DM)	691	680	691
Palmitic acid, g/100 g	10.7	9.43	12.9
Stearic acid, g/100 g	4.08	6.11	4.48
Oleic acid, g/100 g	31.9	32.0	34.4
Linoleic acid, g/100 g	48.2	38.2	44.4
α-Linoleic acid, g/100 g	2.9	11.5	1.8

**Table 2:** Gross chemical composition of semi-hard cheese made of milk from goats fed with diets containing extruded linseed (ELS) or pumpkin seed cake (PSC).

	Diets			SEM	*p*-Value		
	Control	ELS	PSC		D	P	D × P
Moisture, %	53.4 ^a^	51.1 ^c^	52.1 ^b^	0.28	<0.001	<0.001	0.661
Protein, %	21.0	21.0	20.6	0.25	0.128	<0.001	<0.001
Fat, %	23.1 ^b^	24.1 ^a^	24.1 ^a^	0.24	<0.001	<0.001	<0.001
Ash, %	2.04	2.05	2.06	0.013	0.810	0.164	0.505
pH	5.85 ^b^	5.90 ^a^	5.85 ^b^	0.014	0.016	<0.001	<0.001
Fat/protein	1.12 ^b^	1.15 ^a, b^	1.17 ^a^	0.022	0.001	<0.001	<0.001

D = diet effect; P = period effect (20th, 48th, and 75th day of the study period); SEM = standard error of the mean. ^a, b, c^ Values within a row with different superscripts differ significantly at *p* ≤ 0.05.

**Table 3:** Mean values of fatty acid proportions (g/100 g fatty acids) of semi-hard cheese made of milk from goats fed with diets containing extruded linseed (ELS) or pumpkin seed cake (PSC).

g/100 g FAME	Diets			SEM	*p*-Value		
	Control	ELS	PSC		D	P	D × P
C4:0	1.60 ^b^	2.14 ^a^	1.60 ^b^	0.063	<0.001	0.277	0.146
C6:0	1.79 ^a^	1.57 ^b^	1.76 ^a^	0.039	0.012	0.249	0.009
C8:0	2.44 ^a^	2.21 ^b^	2.31 ^a, b^	0.045	0.029	<0.001	0.091
C10:0	9.10 ^a^	8.23 ^b^	8.54 ^b^	0.162	0.002	<0.001	0.011
C12:0	4.42 ^a^	3.74 ^b^	3.93 ^b^	0.088	<0.001	<0.001	<0.001
C14:0	10.5 ^a^	9.11 ^c^	9.95 ^b^	0.169	<0.001	<0.001	<0.001
C15:0	0.980 ^a^	0.893 ^b^	0.904 ^a, b^	0.0303	0.026	<0.001	0.002
C16:0	27.7 ^a^	25.6 ^b^	27.7 ^a^	0.24	<0.001	<0.001	<0.001
C16:1 c9	0.644 ^a^	0.461 ^b^	0.695 ^a^	0.0418	<0.001	<0.001	<0.001
C17:0	0.954 ^a^	0.848 ^b^	0.832 ^b^	0.0212	<0.001	<0.001	<0.001
C18:0	11.8 ^c^	14.7 ^a^	13.5 ^b^	0.32	<0.001	<0.001	<0.001
C18:1 t9	3.10 ^b^	3.24 ^a^	2.79 ^b^	0.176	<0.001	<0.001	<0.001
C18:1 c9	19.5 ^b^	21.4 ^a^	20.1 ^b^	0.35	<0.001	<0.001	<0.001
C18:2 c9t11	0.310 ^b^	0.389 ^a^	0.318 ^b^	0.0152	<0.001	<0.001	<0.001
C18:2 n-6	3.06 ^a, b^	2.84 ^b^	3.20 ^a^	0.105	0.043	<0.001	<0.001
C18:3 n-3	0.535 ^b^	0.833 ^a^	0.458 ^c^	0.0327	<0.001	<0.001	<0.001
C20:0	0.292 ^b^	0.318 ^a^	0.282 ^b^	0.0073	<0.001	<0.001	0.032
C20:4	0.259 ^a^	0.209 ^b^	0.210 ^b^	0.0079	<0.001	<0.001	<0.001
C20:5 n-3	0.097	0.093	0.088	0.0055	0.623	<0.001	0.007
C21:0	0.890 ^b^	1.10 ^a^	0.875 ^b^	0.0408	<0.001	<0.001	<0.001

D = diet effect; P = period effect (20th, 48th, and 75th day of the study period); SEM = standard error of the mean. ^a, b, c^ Values within a row with different superscripts differ significantly at *p* ≤ 0.05.

**Table 4:** Mean values of groups of fatty acid proportions (g/100 g fatty acids) in semi-hard cheese of goats fed with diets containing extruded linseed (ELS) or pumpkin seed cake (PSC).

g/100 g FAMEs	Diets			SEM	*p*-Value		
	Control	ELS	PSC		D	P	D × P
SCFAs	14.9	14.2	14.2	0.24	0.092	<0.001	0.008
MCFAs	45.2 ^a^	40.7 ^c^	44.0 ^b^	0.46	<0.001	0.060	<0.001
LCFAs	32.9 ^c^	45.2 ^a^	41.8 ^b^	0.58	<0.001	0.060	<0.001
SFAs	72.6 ^a^	70.2 ^b^	71.9 ^a^	0.31	<0.001	<0.001	<0.001
MUFAs	23.3 ^b^	25.2 ^a^	23.6 ^b^	0.30	<0.001	<0.001	0.004
PUFAs	3.63	4.44	4.25	0.123	0.536	<0.001	0.018
n-3	0.632 ^b^	0.928 ^a^	0.547 ^c^	0.032	<0.001	<0.001	<0.001
n-6	3.63	3.43	3.73	0.103	0.115	<0.001	<0.001
n-6/n-3	5.71 ^b^	3.80 ^c^	6.89 ^a^	0.248	<0.001	0.816	0.003
AI	2.70 ^a^	2.26 ^c^	2.57 ^b^	0.052	<0.001	0.013	<0.001
TI	3.23 ^a^	2.86 ^b^	3.32 ^a^	0.044	<0.001	<0.001	0.001
HPI	0.37 ^b^	0.45 ^a^	0.39 ^b^	0.010	<0.001	0.022	<0.001

D = diet effect; P = period effect (20th, 48th, and 75th day of the study period); FAMEs = fatty acid methyl esters; SCFAs = short-chain fatty acids; MCFAs = medium-chain fatty acids; LCFAs = long-chain fatty acids; SFAs = saturated fatty acids; MUFAs = monounsaturated fatty acids; PUFAs = polyunsaturated fatty acids; AI = atherogenic index; TI = thrombogenic index; HPI = health promoting index. SEM = standard error of the mean. ^a, b, c^ Values within a row with different superscripts differ significantly at *p* ≤ 0.05.

## Data Availability

The data are available upon request.
